# Direction of Biological Motion Affects Early Brain Activation: A Link with Social Cognition

**DOI:** 10.1371/journal.pone.0131551

**Published:** 2015-06-29

**Authors:** Alan John Pegna, Elise Gehring, Georg Meyer, Marzia Del Zotto

**Affiliations:** 1 University of Geneva, Faculty of Psychology and Educational Science, Geneva, Switzerland; 2 Laboratory of Experimental Neuropsychology, Neuropsychology Unit / Neurology Clinic, Geneva University Hospital, Geneva, Switzerland; 3 University of Liverpool, Dept of Psychological Sciences, Eleanor Rathbone Building, Liverpool, United Kingdom; University of Tuebingen Medical School, GERMANY

## Abstract

A number of EEG studies have investigated the time course of brain activation for biological movement over this last decade, however the temporal dynamics of processing are still debated. Moreover, the role of direction of movement has not received much attention even though it is an essential component allowing us to determine the intentions of the moving agent, and thus permitting the anticipation of potential social interactions.

In this study, we examined event-related responses (ERPs) in 15 healthy human participants to light point walkers and their scrambled counterparts, whose movements occurred either in the radial or in the lateral plane. Compared to scrambled motion (SM), biological motion (BM) showed an enhanced negativity between 210 and 360ms. A source localization algorithm (sLORETA) revealed that this was due to an increase in superior and middle temporal lobe activity. Regarding direction, we found that radial BM produced an enhanced P1 compared to lateral BM, lateral SM and radial SM. This heightened P1 was due to an increase in activity in extrastriate regions, as well as in superior temporal, medial parietal and medial prefrontal areas. This network is known to be involved in decoding the underlying intentionality of the movement and in the attribution of mental states. The social meaning signaled by the direction of biological motion therefore appears to trigger an early response in brain activity.

## Introduction

Biological motion (BM) has received much attention over the last decades and a number of studies have demonstrated that moving organisms are processed rapidly and efficiently. In fact, just a few points of light placed at the principle joints of a moving body suffice for a viewer to immediately recognize that the pattern of motion belongs to an otherwise invisible human being [1] and allows one to identify the type of action being executed, the direction of motion [1,2,3], in addition to attributes such as for example the gender [4,5] or the emotional state of a walker [2,6,7].

The direction of motion of a point-light walker is easy to identify when it occurs in the frontoparallel plane, this is not the case when the point-light walker faces the viewer. Indeed, in the absence of any visual cues indicating perspective, a point light walker facing an observer is ambiguous and can be interpreted as walking either towards or away from the viewer. Interestingly however, rather than being equally distributed as could be expected, frontal views of point-light walkers are more often perceived as walking *towards* the viewer [8], and even more so if the walkers are male [9,10]. This so-called facing bias, it has been argued, may result from the fact that an approaching walker is socially and biologically relevant, and the cost of not detecting an approaching walker is higher than that of missing a walker oriented in another direction [9,11,12], particularly if the walker constitutes a potential threat. Although subsequent findings suggested that this effect was due at least partly to specific patterns of movement occurring in the lower portions of the point light walkers [13], others have pointed out that top down factors such social anxiety also affect the viewers’ perception of an in-depth walker [11,12], illustrating the importance of considering BM not just from its visual perspective, but also in terms of its social significance.

The case for a strong link between BM and social neuroscience has recently been made by Pavlova [14]. The author reviewed evidence regarding BM processing in neurodevelopmental disorders such as autism, fragile X syndrome, Down syndrome and Williams syndrome, which produce different patterns of deficits in social interaction. Pavlova argued that deficits in BM processing were related to those in social cognition in these pathologies. Since BM and social cognition also recruit common brain structures, this suggests a strong relation between the two abilities.

Current evidence therefore suggests that biological movement in the depth plane is highly relevant from a social and biological perspective. Yet nothing is known of the dynamics of brain activation in these conditions. Event-related potential (ERP) studies in healthy human participants have attempted to identify the temporal dynamics of BM processing but have not examined the role of direction of movement. Generally, BM has been investigated by comparing point light stimuli with scrambled versions of the same stimuli (scrambled motion-SM-) and observing differences in the time course of the neural response between the two. In two separate studies [15,16], the earliest ERP difference between BM and SM was found at 200 ms, the former producing a more pronounced negative peak in the occipito-temporal region than the latter. The negativity was then maintained for over 200ms, with a second peak appearing at 240 ms in the earlier study, and at 300ms in the later one. Jokisch, Daum et al. [17] confirmed the presence of a negativity over the 230–360ms time window for BM, also evidencing a right lateralization of the response. Finally, even in 8 month-old infants, a similar time window was identified [18,19] when comparing point light animations with the inverted version of the display, suggesting that BM is processed early in life.

Evidence therefore points to a specific time window for neural processing of biological motion, but the influence of direction, in particular lateral vs. radial motion and its implicit significance on a social level, has not been specifically investigated. We therefore carried out this study with the dual aim of confirming the time course of brain activation for BM, but more importantly, of ascertaining the effect of direction of BM on the electrical response. BM was considered to convey social intent when radial movements of a point light walker were presented, while lateral movements were considered less indicative of any upcoming social interaction. We thus presented human point light displays and their scrambled variants moving in these different planes in order to establish how these two variables (BM and plane of movement) affected the ERPs.

## Method

### Participants

Seventeen volunteers (9 females, mean age = 23.7, SD = 2.02) took part in the experiment, two subjects were subsequently rejected due to excessive artifacts in the EEG signal. All participants were right-handed according to the Edinburgh Handedness Inventory [20], and all had normal or corrected-to-normal vision. They were paid for their participation and gave written consent prior to the study which was approved by the ethics committee of the Faculty of Psychology and Educational Science of the University of Geneva.

### Procedure and Stimuli

**Fig 1:**
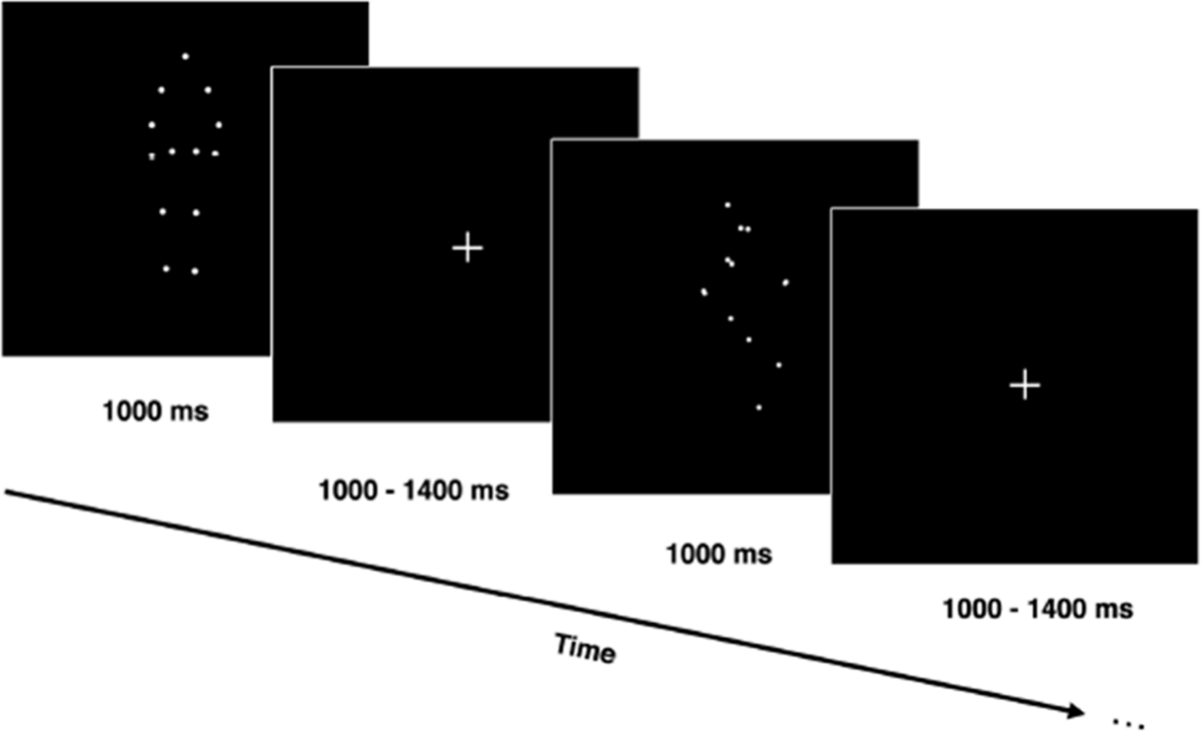
Experimental procedure. Moving stimuli were presented for 1000 ms, followed by a fixation cross at the center of the screen with a variable duration (between 1000 ms and 1400 ms) before the presentation of the next stimulus. Subjects were instructed to press a key according to the direction of the cyclist alone. Only the walker and the cyclist are shown here.

Participants were presented with three types of point-light display stimuli: a cyclist, a walker and a scrambled stimulus that appeared in equal proportions. All stimuli moved either in the radial plane (including forward and backward motion), or in the lateral plane (including right and left motion). The behavioural task was to detect the direction of the cyclist. Half the participants were asked to press one key on the keyboard when the cyclist was moving laterally and another key when the cyclist was moving radially. The keys were reversed for half the subjects. Participants were not required to respond to the walker or the scrambled stimuli. The stimuli were shown for 1000 ms, followed by a fixation cross with a duration randomly varying between 1000 and 1400 ms (Fig 1).

A small number of trials were presented as a training block to ensure the task was understood. The experiment consisted of 7 presentation blocks of 144 trials each (24 repetitions X 6 conditions in each block) for a total of 168 repetitions in each experimental condition. The blocks were presented with E-Prime 2.0 (Psychology Software Tools, Pittsburgh, PA). The stimuli were created using the spatial coordinates of the “Walk” and “Cycle” action files from the Action Database [8] in which 13 dots are used to represent the human body. Matlab (http://www.mathworks.com/products/matlab/) was then used to generate the movie sequences of 1000 ms with a frequency rate of 30 Hz. The dots were white on a black background. The scrambled stimuli were generated from the “Walk” coordinates, by randomly translating the starting position of each point within the walker-defined area. The translation applied to the first frame for a specific point was applied to all the subsequent frames in order to keep the same total momentum. We used two different stimulus sizes to control for a potential size effect. Indeed, the stimuli moving radially change in size over time. Since the EEG analysis is highly time specific, it is possible that the change in stimulus size over time could confound the recordings. Since change in size is necessarily linked to the direction of radial motion, we decided to address this issue by using two different absolute stimulus sizes. This allowed us to have onset, as well as offset sizes, of 3° whether for approaching or receding motion. For lateral motion, an angular size of 2° was employed for the small and 4° for the big size. The left and right stimuli produced the appearance of movement to the left or right although the stimuli at the center of the screen, as if moving on a treadmill. In order to keep the same mean sizes in the approaching/receding stimuli as in the left and right ones, the former were created so as to increase from 1° to 3° (or decrease from 3° to 1°) for the small size, and to increase from 3° to 5° (or decrease from 5° to 3°) for the big size.

Subsequently, a point-wise paired t-test was carried out comparing the ERPs for the “small” and “big” stimuli in each experimental condition using a threshold of p<.05 (corrected for the number of electrodes). Differences were considered significant if the threshold was reached for more than 10ms in more than 3 contiguous electrodes [21]. As no differences were observed, the ERPs for small and big stimuli were consequently merged for the remaining analyses.

### EEG Recordings and Processing

Participants were comfortably seated in an electrically isolated room and situated 120 cm from a 17” display (resolution: 1024 x 768, refreshing rate: 60 Hz) for the experiment presentation. Continuous EEG was recorded at 1024 Hz using the 64-channel BioSemi Active Two system (Biosemi Inc, Amsterdam, Netherlands). The signal was bandpass filtered on-line between 0.1 Hz and 100 Hz. This system uses a Common Mode Sense (CMS) electrode located over the vertex as the reference electrode. Impedances were checked and maintained below 20 kΩ at the onset of the recording. Automatic artifact rejection was performed for epochs containing amplitudes that exceeded ±100 μV and a manual screening was performed to remove any trials containing transients or eye-blinks. The average percentage (± standard error of the mean) of artifact-free epochs was 80.51 ± 10.05 for radial-SM condition, 81 ± 9.51 for lateral-SM condition, 80.3 ± 9.2 for radial-BM condition, and 79.61 ± 10.21 for lateral-BM condition. Bad electrodes were removed and re-interpolated using 3D splines [22].

The signal was filtered offline with the Cartool software (http://brainmapping.unige.ch/cartool.htm version 3.52; release 2507) from 0.17 Hz to 30 Hz and recalculated against the average reference. Epochs of 100 ms pre- and 1000 ms post-stimulus onset, were used to average the ERPs, as well as for the baseline correction.

## EEG Data Analysis

### ERPs

#### Component analysis

The cyclist used as the target was not considered in the analysis. ERPs for incorrect responses were excluded (i.e., when the participant responded with a key press to the walker or scrambled stimuli).

The latencies and amplitudes of the P1 and N1 peaks were investigated by determining the maxima within the 80 ms–150 ms and 170 ms–200 ms time periods respectively. The mean amplitude for an early sustained negativity (ESN) was computed over the 210 ms–360 ms time window.

ANOVAs for repeated measures were conducted using Motion (biological, scrambled) X Direction (Radial, Lateral) X ROI (Left, Right, [Central]) as factors. We applied the Greenhouse-Geisser correction when necessary. Based on visual observation of the maximum ERP effects, 2 ROIs were created for the P1 and N1 components, one on the left (P5, P7, PO7) and the other on the right (P6, P8, PO8). Three ROIs were created for the ESN: left (P9, P7, P5, PO7), central (O1, Oz, O2, Iz), and right (P10, P8, P6, PO8).

#### Topographic segmentation analysis

In order to identify whether any differences across conditions were due to differences in the spatial configuration of the scalp topographies, a topographic segmentation analysis was performed on the grand mean ERPs of the 4 conditions (SM lateral, SM radial, BM lateral, BM radial).

This method has been described in detail elsewhere [23,24]. Briefly, it examines the succession of electrical scalp potential maps constituting the ERPs in the different conditions and determines the time periods during which these maps remain stable. Differences in the topographies of the scalp potential maps are thought to reflect differences in the underlying neuronal generators, and it has been suggested that the periods of stable topographies correspond to specific steps in information processing during which a given neural network configuration is active. These periods have consequently been termed functional microstates [25,26,27,28]. These functional microstates are determined by template maps that are extracted by means of a spatial k-means cluster analysis that identifies the dominant map topographies in the grand average ERPs across the experimental conditions over time [27,28]. The smallest set of maps that accounts for the greatest amount of variance is then selected using a cross validation criterion [24,29,30,31,32,33].

### Source Localization Analysis

In order to estimate the cerebral regions giving rise to the differences in processing for BM/SM and radial/lateral directions, we applied a standardized low-resolution tomography algorithm-sLORETA- (http://www.uzh.ch/keyinst/loreta.htm [34]) on the time periods producing ERP differences. LORETA (Low Resolution Electromagnetic Tomography) is an algorithm that solves the inverse problem by computing the smoothest of all possible distributions of current density, i.e, by minimizing the norm of the Laplacian of the 3D current distribution [34]. Using a three-shell head model, sLORETA is computed on 6239 voxels placed within the cortical and hippocampal grey matter of the Montreal Neurological Institute's (MNI) reference brain with a spatial resolution of 5mm.

Statistical computations were performed on the current density data of the whole-brain sLORETA solutions. Using paired-samples t-tests comparisons were performed between conditions on the periods that were significant in the ERP analysis. A single classical t-test was computed for averaged voxels/nodes in the windows corresponding to the ERP components based on 5000 SnPM randomizations with bullet proof in order to correct critical thresholds and p-values.

## Results

The mean correct response rate was of 98.6%, showing that participants were actively engaged in the task and that it was easy to perform.

### ERP components

**Fig 2:**
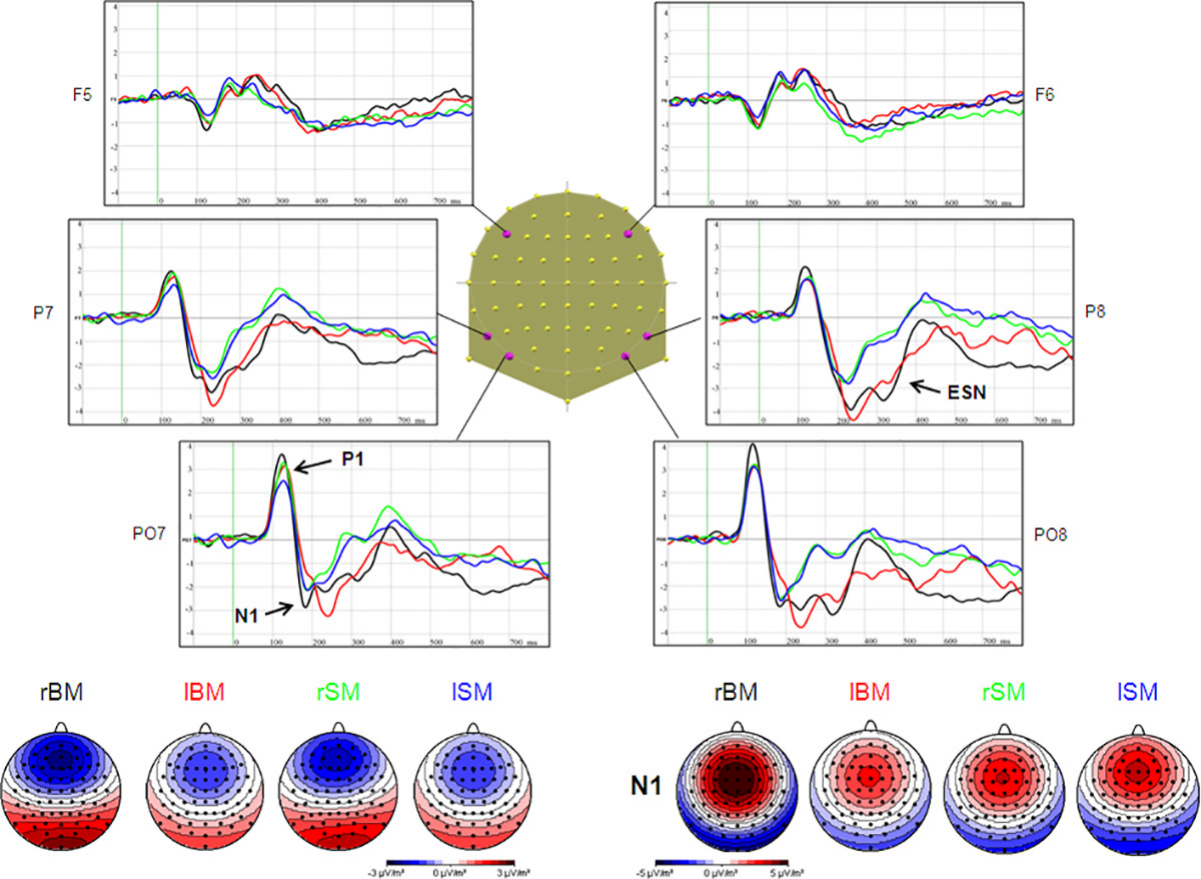
ERPs and scalp current density maps of the four different conditions. Radial (rBM in black) and lateral (lBM in red) biological motion, as well as radial (rSM in green) and lateral (lSM in blue) scrambled motion are represented over 6 electrodes (4 posterior and 2 anterior). Below: scalp current density maps of P1 (right) and N1 (left) components, representing the four conditions, are computed on the mean between 170 ms and 200 ms, and between 80 ms and 150 ms respectively.

As expected, the P1 appeared as a positive deflection over posterior electrodes bilaterally peaking at around 115ms. This was followed by a bilateral negativity over posterior electrodes at around 190ms, the N1 component. Following the N1, the posterior leads showed a second sustained negative potential (that we called the *early sustained negativity*–ESN–) until almost 400ms that was more marked for biological compared to scrambled motion. During this period, a progressive shift to more positive potentials appeared simultaneously over central leads. Fig 2 illustrates the ERPs at 3 left and 3 right electrodes for BM and SM each in the 2 planes of movement.

#### P1

*Amplitude*: Mean and standard error of the mean (SEM) for each condition are: BM radial: 2.27 μV ± 0.35, BM lateral: 1.75 μV ± 0.26, SM radial: 1.81 μV ± 0.27, SM lateral: 1.84 μV ± 0.32.

A 2 X 2 X 2 ANOVA, including motion (BM or SM), direction (radial or lateral) and ROI (left or right) was computed on the peak amplitudes values and revealed a significant effect of direction (F_(1, 14)_, ε = 1, *p* < 0.04) as well as a motion—direction interaction (F_(2,28)_ = 4.72, ε = 1, *p* < 0.05). This is due to the fact that P1 is greater for radial motion but only when the movement is biological (post-hoc Tukey HSD: *p* < 0.05), while this effect is not observed for SM (*p* > 0.9).

A similar 2 X 2 X 2 ANOVA at P1 latencies showed an effect of motion (F_(1,14)_ = 5.32, ε = 1, *p* < 0.04), with an earlier peak for biological (112ms ± 3.18) compared to scrambled (118ms ± 3.08) (Fig 2).

#### N1

The 2 (motion) X 2 (direction) X 2 (ROI) ANOVA revealed a significant effect of Motion (F_(1,14)_ = 5.85, ε = 1, *p* < 0.03) due to a greater amplitude for BM (-2.84 μV ± 0.56) compared to SM (-2.41 μV ± 0.46), as well as a Direction effect (F_(1,14)_ = 20.59, ε = 1, *p* < 0.001) due to a greater amplitude for radial (-2.93 μV ± 0.50) compared to lateral motion (-2.32 μV ± 0.52).

The statistical analysis of the latency revealed no significant effects.

#### ESN (210–360ms)

As for the N1, the 2 (motion) x 2 (Direction) x 3 (ROIs) ANOVA performed on the mean amplitude over the 70ms time window yielded a significant effect of motion (F_(1,14)_ = 62.82, ε = 1, *p* < 0.001) (BM: -3.74 μV ± 0.44, SM: -2.18 μV ± 0.32) with a globally more negative ESN for BM than SM. In addition, a significant motion—ROI interaction (F_(2,28)_ = 13.48, ε = 0.99, *p* < 0.001) was found due to a greater difference in the right ROI between BM and SM (*p* < 0.001 on the post-hoc Tukey HSD).

To summarize, the P1 produced greater amplitudes for radial biological motion. The N1 also showed a stronger negativity for radial movement independently of the type of motion (BM or SM). On the other hand, the N1 and ESN showed greater amplitudes for biological motion in general, independently of direction.

#### Approaching vs. receding motion

Since both the P1 and the N1 components were sensitive to radial compared to lateral motion, an additional comparison was performed to establish further whether approaching and receding stimuli differed at these components on the same electrodes. We therefore carried out a 2 (approaching BM vs. receding BM) x 2 (ROIs) ANOVA on the peak amplitudes of the P1 and N1.

No effect of the type of direction was observed on the P1 (F(_1,14_) = 0.99, ε = 1, p > 0.34; mean values: receding = 1.98 μV, ± 0.6 vs. approaching = 2.2 μV, ± 0.68). By contrast the N1 showed a more negative amplitude for receding than looming movements (F(_1,14_) = 5.94, ε = 1, p < 0.03; mean values: receding = -3.75 μV, ± 0.5 vs. approaching = -3.2 μV, ± 0.5).

### Topographic segmentation

**Fig 3:**
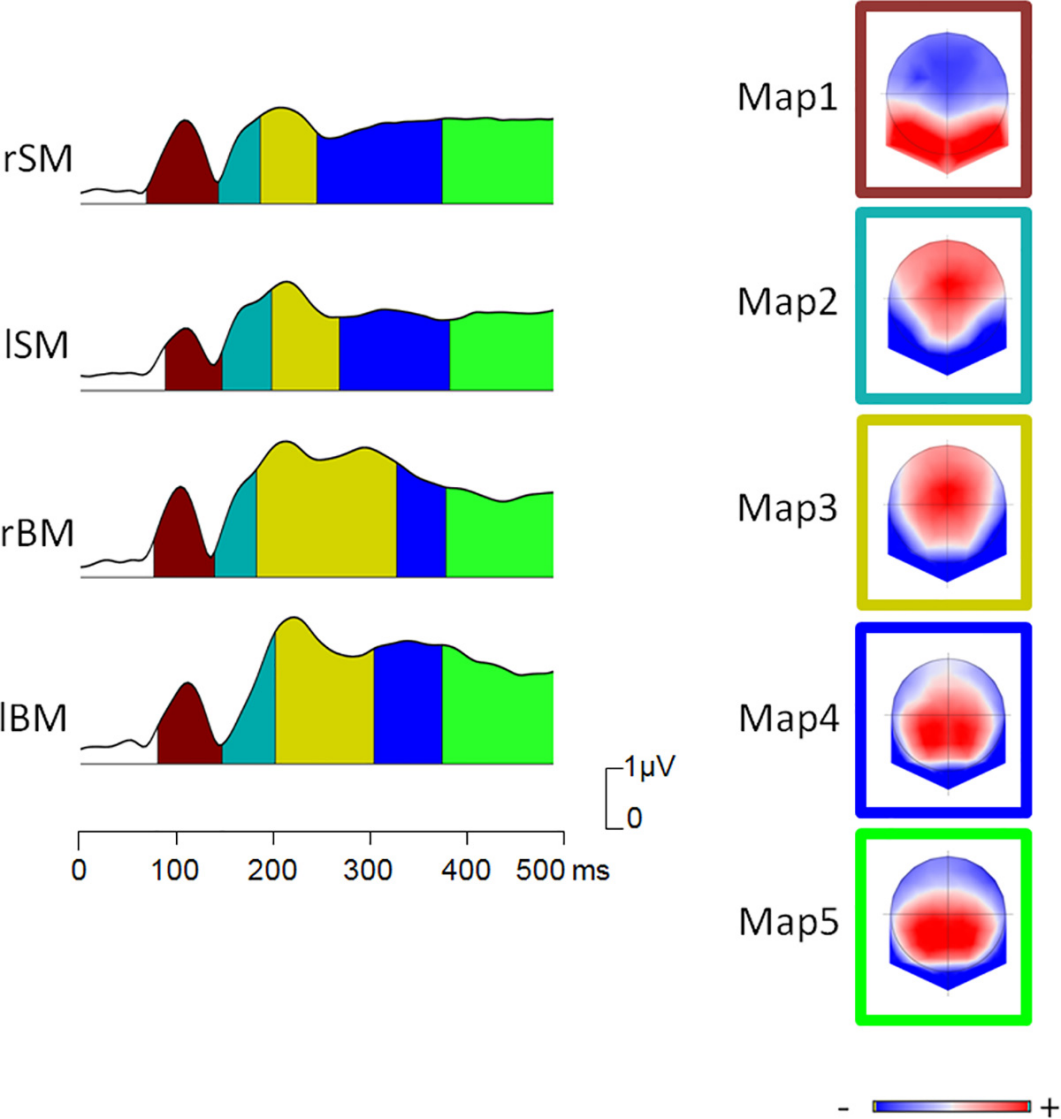
Topographic segmentation. The global field power (GFPs) of the grand average ERPs are shown for radial and parallel motion in the scrambled motion (respectively rSM and lSM) and biological motion conditions (respectively rBM and lBM) over the first 500ms. The topographic segmentation procedure performed on rSM, lSM, rBM and lBM together identified 5 segment maps that best explained the map series of the 4 conditions. These 5 segment maps, numbered Map1 to Map5 are represented on the column on the right and are outlined with different colors. Their times of appearance are indicated by the corresponding color in the area below the GFP in each of the 4 conditions.

A topographic segmentation was performed on the map series of the 2 BM and 2 SM conditions between 80ms and 500ms after stimulus presentation, in order to determine the map topographies that were common or different to the conditions. Five identical topographies were found to best explain the ERP map series in all 4 conditions. The corresponding segment maps (Map1 to Map5) are show in Fig 3 and are represented under the global field power traces of each of the 4 conditions.

All 4 conditions gave rise to the same topographies for the P1 and the N1, these are represented in Fig 3 as segments Map1 and Map2 respectively. The 3 subsequent segment maps encompassing the period of the ESN were also identical on all 4 conditions revealing that the ERP differences were not due to the activation of different neural networks. Of particular interest is the fact that the N1 topography (Map2) differed from the subsequent, ESN topographies (Map3, Map4), justifying their differentiation in the component analysis.

### Source Localization Analysis

Based on the ERP results, we performed a t-test comparing the solutions of different ERPs components between radial and lateral BM conditions, biological and scrambled conditions, as well as radial and lateral motion conditions. Here we report only results that are significant at a threshold of p<.05 in at least 30 adjacent voxels.

#### P1

**Fig 4:**
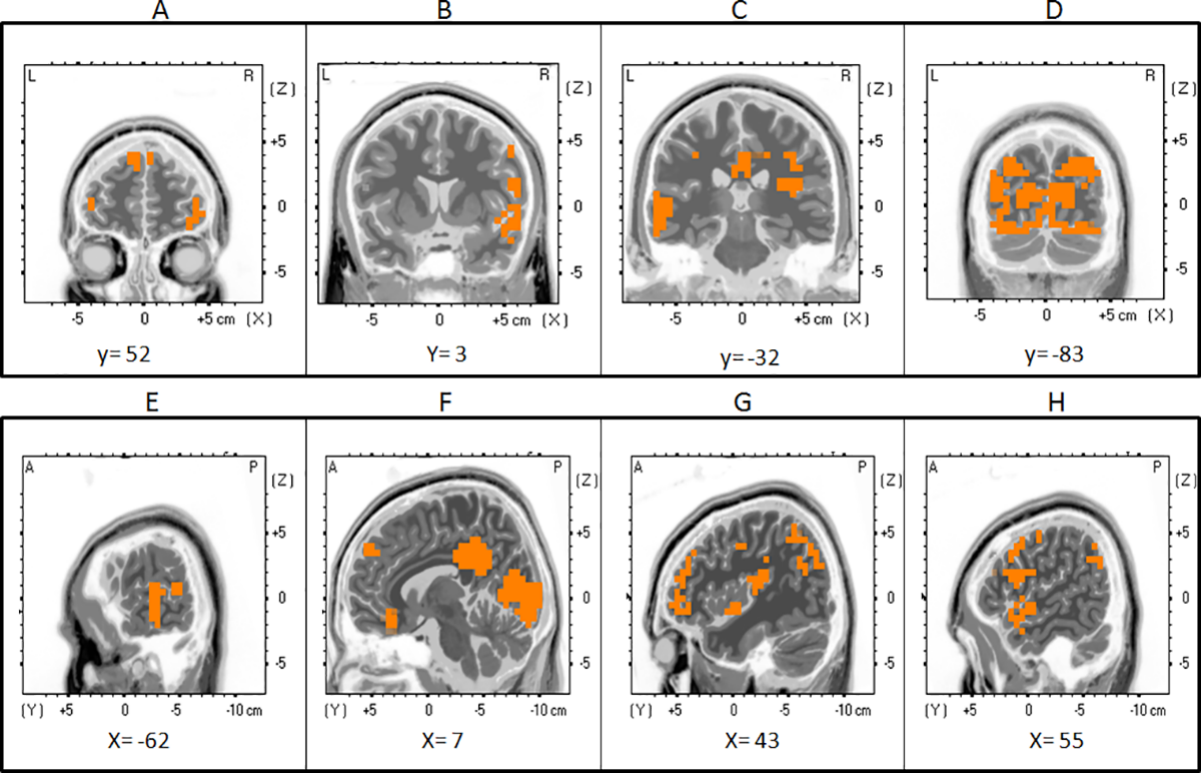
Source Localization for P1. Coronal (top row) and sagittal (bottom row) views of an average brain showing regions of significant cortical activation. A t-test comparing the voxels activated in the source localization analysis (sLoreta) of the P1 component during lateral and radial biological motion was performed. Significant voxels are shown in orange [T(15) = 1.66, p<.05], (Talairach coordinates; MRI template—Colin27 T1). Top row: activation can be seen in the left and right middle and superior frontal gyri (A), the right temporal areas (middle and superior temporal gyri) (B), temporal and limbic areas (left middle and superior temporal gyri, cingulate gyri) (C), as well as occipital regions (cuneus, fusiform gyri, lingual gyri, middle occipital gyri) (D). Bottom row: significant activation is observed in the left middle and superior temporal gyri (A); right superior and middle frontal gyri, cingulate and lingual gyri, cuneus (B); right middle and superior frontal gyri, superior temporal gyrus (C); right middle and temporal gyri and precentral gyrus (D).

As shown in the peak analysis of the P1 component, its amplitude was significantly greater for radial compared to lateral direction only in biological motion condition. The paired comparison between those two conditions at the P1 revealed the existence of several significant clusters situated in different areas (Table 1 and Fig 4). The major clusters were located in the occipital cortex (BA 17, 18, 19), including more specifically parts of the cuneus, [Maximum at Talairach coordinates (TcMax): 30x; −81y; 32z; T_15_ = 5.48, p = .002], the middle occipital gyrus (Max: 30x; −77y; 22z; T_15_ = 3.03, p = .025) and the lingual gyrus (TcMax: -15x; −78y; 4z; T_15_ = 2.9, p = .025). In the temporal lobe, greater activations of adjacent nodes were located in the middle and superior temporal gyri (BA 19, 21, 39; TcMax: 35x; −81y; 27z; T_15_ = 3.36, p = .04), and in the parietal lobe (BA 3, 7, 19, 31) the major activated structures encompassed the precuneus (TcMax: 25x; −66y; 36z; T_15_ = 3.64, p = .04). Two other significant clusters included a large portion of the middle frontal gyrus (TcMax: 50x; 17y; 41z; T_15_ = 2.62, p = .0054) in the frontal pole, and the cingulate gyrus (TcMax: 10x; -42y; 34z; T_15_ = 3.25, p = .04).

**Table 1:** Source localization for P1.

Lobe	Structure	BA
Frontal	Middle Frontal Gyrus (rh, lh)	9, 10, 11, 25, 46, 47
Limbic	Cingulate Gyrus, Posterior Cingulate (rh, lh)	23, 30, 31, 32,
Occipital	Cuneus, Fusiform Gyrus, Lingual Gyrus, Middle Occipital Gyrus, (rh, lh)	7, 17, 18, 19, 23, 30
Parietal	Inferior Parietal Lobule, Precuneus (rh, lh);	7, 19, 31, 39, 40,
Temporal	Middle Temporal Gyrus, Superior Temporal Gyrus (rh, lh)	19, 21, 22, 38, 39, 41, 42

Brain regions (lobes and corresponding Brodmann areas) that showed a significantly greater activation for radial BM than parallel BM for the P1 component.

#### ESN

**Fig 5:**
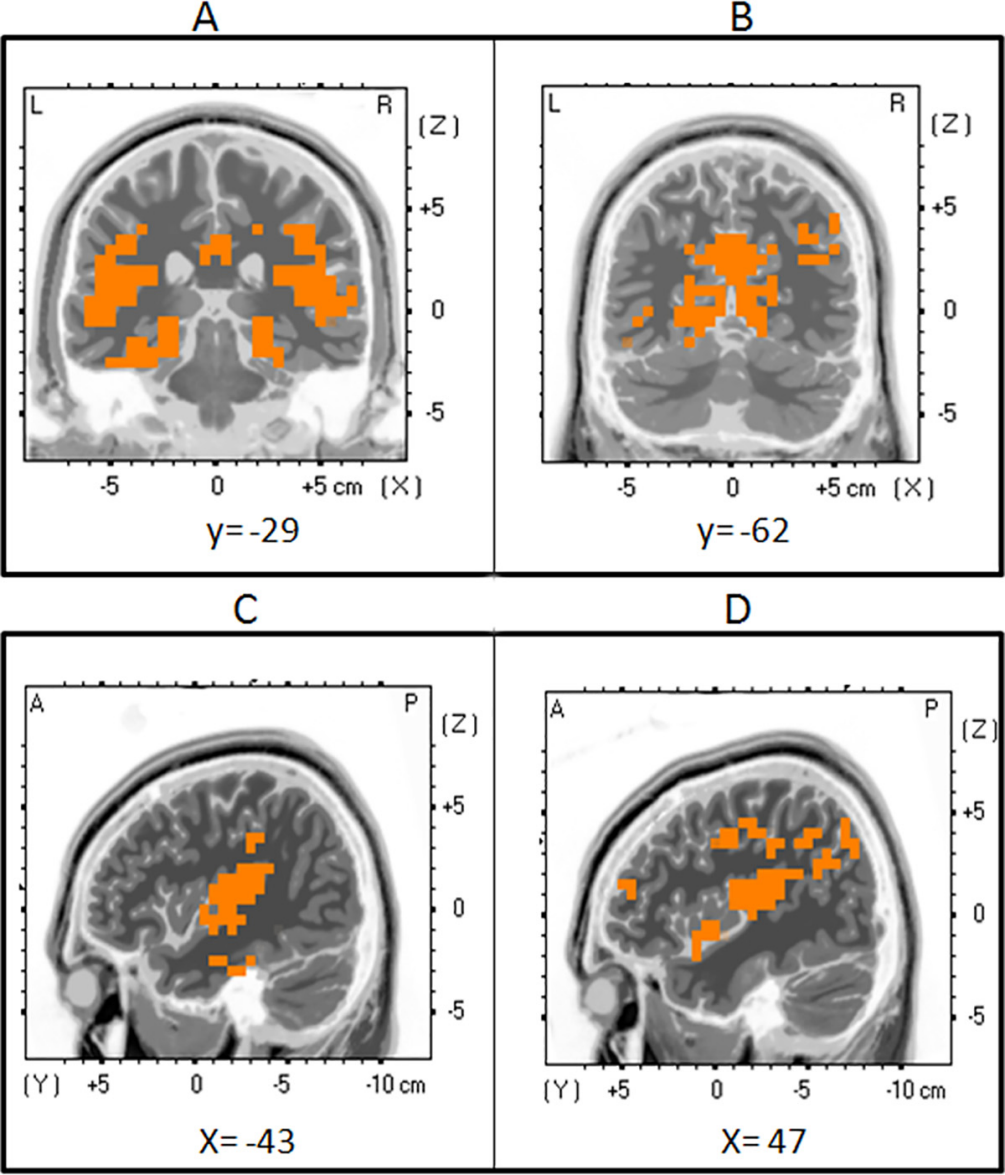
Source Localization for ESN. Coronal (top row) and sagittal (bottom row) views of an average brain showing regions of significant cortical activation. A t-test comparing the voxels activated in the source localization analysis (sLoreta) of the ESN component during biological and scrambled motion was performed. Significant voxels are shown in orange [T(15) = 2.1, p<.01], (Talairach coordinates; MRI template—Colin27 T1). Top row: significant activation is found in left and right temporal and limbic areas (superior temporal gyri, cingulate gyri, parahippocampal gyri, insula) (A); left and right occipital and parietal areas (lingual gyrus and precuneus) regions (B). Bottom row: brain activation is observed in the left superior and transverse temporal gyri and insula (A); right superior temporal gyrus, middle frontal gyrus, precentral and postcentral gyrus, inferior parietal lobe, angular gyrus and insula.

The t-test between biological and scrambled motion showed a strong activation in the limbic lobe for the cingulate gyrus, parahippocampal gyrus and posterior cingulate (TcMax: 0x; -47y; 25z; T_15_ = 5.27, p < .0001) and the insula (TcMax: -30x; -28y; 20z; T_15_ = 4.23, p < .0001). In the temporal lobe, the major group of clusters was located in the superior temporal gyrus (TcMax: 40x; -57y; 30z; T_15_ = 4.66, p < .0001); whereas, in the occipital and parietal lobes, the lingual gyrus (TcMax: -10x; -53y; 3z; T_15_ = 3.85, p < .0001) and the precuneus (TcMax: 0x; -52y; 30z; T_15_ = 5.25, p < .0001) were the structures most activated (Table 2 and Fig 5).

**Table 2:** Source localisation for ESN.

Lobe	Structure	BA
Limbic	Cingulate Gyrus, Parahippocampal Gyrus, Posterior Cingulate, Precuneus (rh, lh)	19, 23, 24, 27, 28, 29, 30, 31, 32, 34, 35, 36
Occipital	Lingual Gyrus (rh, lh)	18, 19,
Parietal	Precuneus (rh, lh)	7, 19, 31
Sub-lobar	Insula (rh, lh)	13
Temporal	Superior Temporal Gyrus (rh, lh)	13, 22, 38, 41

Source Localization for ESN. Brain regions (lobes and corresponding Brodmann areas) that showed a significantly greater activation for BM than SM in the ESN time window.

## Discussion

In this study, we used EEG to investigate the dynamics of brain activation for biological movements compared to scrambled motion, and explored the differences linked to the plane of movement (radial vs. lateral). We found that BM produced a greater N1 and further observed that the increased negativity was maintained between 210 and 360ms in what we call here the early sustained negativity. More importantly in this study, biological motion showed a differential response depending on the plane of movement, with radial BM producing an enhanced P1 compared to the 3 other conditions.

### BM versus SM

The effect of BM on the ERPs corroborates several previous observations. As noted above, the presence of a long, negative going potential in response to biological motion was reported by Hirai et al. [15] who showed a difference between BM and SM processing that appeared as a negative wave peaking at 200ms and again at 240 ms. Subsequently, Hirai et al. [16] again demonstrated the presence of two peaks, culminating this time at 200ms and 330ms. The later peak, which the authors called the N330, was greater when participants attended to the BM stimuli. The N330 was also found to be greater over the right leads, in keeping with our observations. Two subsequent studies further confirmed a similar negativities, identifying them between approximately 200ms and 350ms [17,35]. Indeed, Jokisch et al. [17], compared upright and inverted point light walkers, as well as scrambled stimuli, and observed a greater negativity between 230 and 360ms for walkers (both upright and inverted) compared to scrambled stimuli. Interestingly, they also observed an N180 that was enhanced for upright walkers compared to inverted or scrambled stimuli. This led them to suggest that the N180 reflected the processing of the familiar body shape while the later components was linked to the processing of biological patterns of movement. This conclusion is warranted by several ERP investigations on human body perception that have provided evidence of an enhanced N1 for human bodies [36,37,38,39,40]. Of particular relevance here, Thierry et al. [40] revealed the existence of a body-sensitive N190 that appeared not only in response to photographs and silhouettes of human bodies, but also to stick figures that are closer to the stimuli used in our study.

Our findings of an N1 followed by an ESN are in line with these previous observations of two negative components. In our case, this later negativity did not possess a clearly identifiable peak leading us to use the term *sustained* negativity. These two periods are nevertheless clearly different, as highlighted by our topographic segmentation procedure that distinguished the N1 and ESN as distinct scalp topographies, which in turn suggests the activation of dissimilar brain networks for these two periods. In the light of our findings, we would surmise that the N1 might reflect the processing of body shape and of direction of motion as distinct processes that are not yet integrated, in view of the fact that each process was significant as a main effect. ESN on the other hand appears to reflect the processing of biological movement per se. However, very recently, White et al. [41] contested the idea of a specific ERP marker for biological motion. In their study, the authors attempted to extricate the role of human form and action perception from biological motion that they suggest were confounded in the preceding studies. Comparing the ERP response to upright, inverted and scrambled point light displays, in addition to stick figures, they found that the effects on the N1 time window were present both for static and moving bodies, but that they were *greater* for scrambled than upright and inverted stimuli in contradiction with previous reports. On the other hand, no components were found to respond specifically to biological motion, thus questioning the existence of an ERP marker for biological motion. The authors therefore concluded that the ERP effects observed in initial studies must in fact reflect the integration of action and form perception rather than biological motion alone, with both processes being closely linked. This conclusion cannot be corroborated by our study, as neither static nor inverted stimuli were included in the experimental paradigm.

Interestingly, an earlier time period for BM has also been suggested in other reports [35,42,43,44]. Using time frequency analysis, Pavlova et al. [44] measured the MEG response while presenting upright and inverted point-light walkers, as well as scrambled stimuli. They found that both upright and inverted walkers evoked an increased gamma-band activity compared to baseline, beginning as early as 80 ms and reaching a maximum at 100 ms after the beginning of the stimulus. On the other hand, upright and inverted scrambled stimuli showed no such effect. In a subsequent study, the authors replicated this effect, further demonstrating that the early response remained even when subjects were not required to attend to the stimuli [43].

Using ERPs, Krakowski et al. [35] evidenced an increased P1 amplitude for biological compared to scrambled motion. Buzzell et al, [42] subsequently confirmed this early response using intact and scrambled point light walkers, but also static versions of the stimuli. In their study, the P1 was enhanced for dynamic and for static point light walkers compared to scrambled stimuli. This suggested that the early response might be related to the extraction of global form information from the stimuli, rather than to BM per se, however, our data failed to show any enhancement of P1 for intact walkers compared to scrambled stimuli and do not corroborate this conclusion.

Our current finding therefore confirms the importance of the ESN as the time window during which biological motion is processed. This interpretation is further strengthened by the sLORETA solutions, which revealed notably an activation of the superior temporal gyrus and superior temporal sulcus when contrasting BM with SM. These anatomic locations agree perfectly with studies using brain imaging techniques that have repeatedly shown the implication of the posterior STS for point light stimuli compared to scrambled or inverted ones [45,46,47,48,49].

### Radial versus Lateral Motion

The main focus of the current study was specifically to investigate whether brain processing was affected by the direction of biological motion (radial or lateral), with radial motion being taken as a sign of intent (approach or withdrawal), as opposed to lateral motion. A possible confound may exist in that radial and lateral motion could produce different neural response profiles occurring independently of intent. However, the earliest effect of direction was seen as an increase in P1 amplitude for radial biological motion, while radial scrambled motion and both lateral movements remained similar. Since radial BM differed from radial SM, it seems unlikely that this early modulation was linked to radial motion per se, but rather it appears to be specifically linked to this plane of motion when it is viewed in conjunction with biological characteristics. Consequently, we would argue that the P1 is indeed specific to biological radial motion, which is distinctive in that it bears socially meaningful information. Along these lines, a previous investigation, focusing on EEG oscillatory suppression, investigated mu oscillation suppression (i.e., 8–12Hz activity in central electrodes) while participants viewed BM [50]. The stimuli that were presented varied across gender, emotion and intention, the latter being determined by the direction of motion (approaching vs. receding) of the walker. Mu suppression was observed in response to BM compared to a control task. Moreover, and of particular interest here, suppression was significantly greater when participants assessed intention, although in this study no difference was reported between approaching and receding stimuli. These findings highlight the link between intention and radial BM, and demonstrate the heightened sensibility of the relevant neural systems to socially meaningful motion.

Our current results thus provide novel experimental evidence that the neural response for radial motion is rapid and enhanced in comparison with lateral motion or with motion that is non-biological, most likely because it conveys information that may be relevant for the viewer’s survival.

The analysis of the P1 generators revealed that radial BM produced a significant increase in activity in the visual striate and extrastriate regions, the fusiform and lingual gyri, the superior and middle temporal gyri, the precuneus and inferior parietal, and finally the prefrontal and cingulate regions. Interestingly, activation of these regions has been observed in studies investigating movements displaying a social component. In fact, Saxe et al. [51] even hypothesized that the superior temporal lobe and STS may be sensitive to the intention of a movement rather than biological motion per se. Using an fMRI paradigm, they presented short video clips of an actor walking across a room. The trajectory of the walker was hidden by an object at which point the actor was hidden from view. Critically, the duration that the walker remained hidden varied, such that longer periods pointed to differences in intentionality. The analysis of BOLD responses, contrasting long durations with short ones, revealed an increase in posterior STS activation, pointing to a role for this structure in understanding the intention of the moving agent. In another study using PET, Castelli et al. [52] presented simple geometrical shapes whose movement patterns evoked relational interactions, or lacked any implication of social behaviour. Their findings revealed activation in the medial prefrontal cortex (including BA 9), the superior temporal sulcus (including 22 and 39), basal temporal regions (including the fusiform gyrus and temporal poles in BA 38), and extrastriate cortex (including BA 18–19), a network that the authors ascribed to the attribution of mental states and that are closely concordant with our findings.

Similarly, an implication of the medial prefrontal cortex, as well as the precuneus was confirmed for social aspects of movement by Iacoboni et al. [53]. They compared the BOLD response of healthy participants watching movie clips that showed either single individuals engaged in particular activities, or two persons interacting. Interaction clips yielded increased activity both in dorsomedial prefrontal as well as in the medial parietal (precuneus) cortices.

A high degree of overlap can be seen between the regions identified in our study and those established in the investigations reported above. We would surmise that the regions activated during the P1, including the medial prefrontal cortex, the superior temporal cortex, the precuneus and the extrastriate areas, all more highly activated for radial movement compared to lateral movement, are in fact involved in the interpretation of meaningful, social aspects of biological motion. Critically, our findings show a rapid and early emergence of this activity in the course of visual processing.

The presence of STS sources during the P1 (i.e., around 115ms) may seem to be somewhat early. Indeed, although intracranial responses of neurons in the STP region of the macaque have been observed at 100ms [54], the size and neural circuits in the human visual system are different from the monkey brain and may give rise to a slower transmission of information down the visual stream [55]. Consequently, human STS activity at 100ms might seem too fast. Of course, as noted above, neural responses specific to biological motion have been reported at these times, and even earlier [35,44], however they did not provide anatomical evidence implicating the STS. The ERP study that reported effects at 100ms for BM identified sources using dipoles [35] and found activity close to hMT, but did not include the STS region. Elsewhere, evidence has emerged showing that information regarding motion can progress quite quickly upstream in neural processing; for instance, an intracranial study in an epileptic patient showed that (non-biological) movement produced a response in the initial portion of the ascending limb of STS [56] that peaked at 130ms. This leaves open the possibility of relatively early responses for biological motion in humans. Nevertheless, it is important to emphasise that the LORETA algorithm produces a blurred solution and does not possess a very high spatial resolution. Therefore, the anatomical locations reported here must be considered with some caution and should not lead to overinterpretation.

## Conclusions

The results of this investigation show that radial biological motion enhances the early P1 component, an effect that is not observed for radial scrambled motion or for lateral biological motion. The LORETA source localization algorithm points to a significant increase in the activity of generators associated with the attribution of mental states and of intentionality during this early stage. On the other hand, biological motion, irrespective of direction, is processed at around 210ms to 360ms seemingly by the superior temporal region. Socially relevant motion thus appears to be processed very early in the course of brain activation.

To summarise, we speculate here that the processing of biological movement is closely intertwined with the processing of the intentions of the moving agent. As directionality contains highly relevant information concerning the objectives and intent of the moving agent, it is an essential element in preparing for interactions with other individuals and therefore possibly threatening situations. We would conjecture that evolution has implemented neural mechanisms that lead to rapid integration of directional information, allowing us to detect the motion of a person along the radial plane and to respond accordingly. Future investigations should include radial motion of non-human biological organisms in order to determine whether these effects are present in social situations alone, or whether they are triggered more generally by the movement, and potential threat, of any living animal.
